# Addendum: Genome evolution and diversity of wild and cultivated potatoes

**DOI:** 10.1038/s41586-022-05298-5

**Published:** 2022-09-13

**Authors:** Dié Tang, Yuxin Jia, Jinzhe Zhang, Hongbo Li, Lin Cheng, Pei Wang, Zhigui Bao, Zhihong Liu, Shuangshuang Feng, Xijian Zhu, Dawei Li, Guangtao Zhu, Hongru Wang, Yao Zhou, Yongfeng Zhou, Glenn J. Bryan, C. Robin Buell, Chunzhi Zhang, Sanwen Huang

**Affiliations:** 1 Shenzhen Branch, Guangdong Laboratory of Lingnan Modern Agriculture, Genome Analysis Laboratory of the Ministry of Agriculture and Rural Affairs, Agricultural Genomics Institute at Shenzhen, Chinese Academy of Agricultural Sciences, Shenzhen, China; 2 Key Laboratory of Biology and Genetic Improvement of Horticultural Crops of the Ministry of Agriculture, Sino-Dutch Joint Laboratory of Horticultural Genomics, Institute of Vegetables and Flowers, Chinese Academy of Agricultural Sciences, Beijing, China; 3 Graduate School Experimental Plant Sciences, Laboratory of Plant Breeding, Wageningen University and Research, Wageningen, The Netherlands; 4 The AGISCAAS-YNNU Joint Academy of Potato Sciences, Yunnan Normal University, Kunming, China; 5 Department of Integrative Biology, University of California Berkeley, Berkeley, CA USA; 6 Cell and Molecular Sciences, The James Hutton Institute, Invergowrie, UK; 7 Center for Applied Genetic Technologies, University of Georgia, Athens, GA USA

**Keywords:** Agricultural genetics, Genomics, Plant breeding, Agriculture, Evolutionary genetics

Addendum to: *Nature* 10.1038/s41586-022-04822-xPublished online 8 June 2022

By analyses of potato pan-genome and transcriptome, we discovered a TCP transcription factor, *Soltu.DM.06G025210*, determining tuber identity, on the basis of which we named this gene *Identity of Tuber 1* (*IT1*).

After our manuscript was accepted in principle, a study by Nicolas et al.^1^ showed that *BRANCHED1b* acts as a tuberization repressor in aerial axillary buds in tetraploid potato. We note that *IT1* and *BRANCHED1b* are the same gene. The name *BRANCHED1b* represents the *A. thaliana* nomenclature system^2^. We chose the name *IT1* because the function of *Soltu.DM.06G025210* in potato had not been reported before and our study suggests that it acts as a key regulator in potato tuberization, a new function that is different from its orthologue in tomato and *Arabidopsis* where it represses branching. Considering the difference in the function, we proposed the new name *IT1* for *Soltu.DM.06G025210*.

Both studies confirmed that IT1/BRANCHED1b interacts with the mobile tuberization inductive signal SP6A and plays an essential role in tuberization. Further investigation is awaited into how the same gene promotes tuberization in underground shoots (stolons) and represses tuberization in aerial axillary buds
