# Supplementary material for: Genome evolution and diversity of wild and cultivated potatoes
Source: Nature. 2022 Jun 8;606(7914):535–41. doi: 10.1038/s41586-022-04822-x (PMC9200641; doi:10.1038/s41586-022-04822-x)
Supplement: Supplementary file 1 — This file includes Supplementary Figs. 1–11. [file 41586_2022_4822_MOESM1_ESM.pdf]

nature portfolio

https://doi.org/10.1038/s41586-022-04822-x

Supplementary information

Genome evolution and diversity of wild and cultivated potatoes

In the format provided by the
authors and unedited

Supplementary Figures

Genome evolution and diversity of wild and cultivated potatoes

Dié Tang[1,8], Yuxin Jia[1,8], Jinzhe Zhang[3,8], Hongbo Li[1,4,8], Lin Cheng[1], Pei Wang[1], Zhigui Bao[1], Zhihong Liu[1], Shuangshuang Feng[2], Xijian Zhu[2], Dawei Li[1], Guangtao Zhu[2], Hongru Wang[7], Yao Zhou[1], Yongfeng Zhou[1], Glenn J. Bryan[5], C. Robin Buell[6], Chunzhi Zhang[1] and Sanwen Huang[1,*]

[1]Shenzhen Branch, Guangdong Laboratory of Lingnan Modern Agriculture, Genome Analysis Laboratory of the Ministry of Agriculture and Rural Affairs, Agricultural Genomics Institute at Shenzhen, Chinese Academy of Agricultural Sciences, Shenzhen, Guangdong 518120, China.

[2]The AGISCAAS-YNNU Joint Academy of Potato Sciences, Yunnan Normal University, Kunming, Yunnan 650500, China.

[3]Key Laboratory of Biology and Genetic Improvement of Horticultural Crops of the Ministry of Agriculture, Sino-Dutch Joint Laboratory of Horticultural Genomics, Institute of Vegetables and Flowers, Chinese Academy of Agricultural Sciences, Beijing 100081, China.

[4]Graduate School Experimental Plant Sciences, Laboratory of Plant Breeding, Wageningen University & Research, PO Box 386, Wageningen 6700 AJ, the Netherlands.

[5]Cell and Molecular Sciences, The James Hutton Institute, Invergowrie, Dundee DD2 5DA, UK.

[6]Center for Applied Genetic Technologies, University of Georgia, 111 Riverbend Rd, Athens GA 30602, USA.

[7]Department of Integrative Biology, UC Berkeley, Berkeley, CA 94707, USA.

[8]These authors contribute equally to this work.

[*]Correspondence to: huangsanwen@caas.cn (S.H.).

Table of Contents

Pages 3-27: Supplementary Figs 1-11
Page 28: Supplementary References

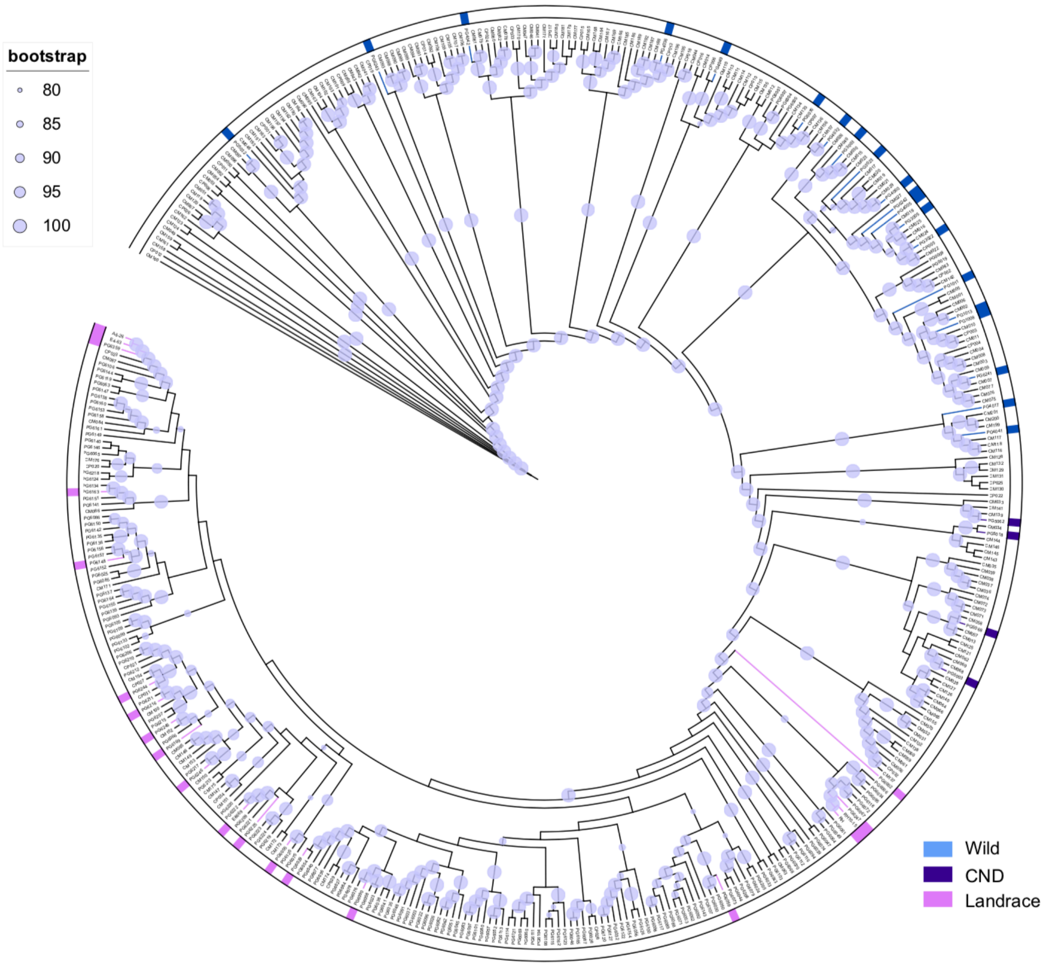

Supplementary Fig. 1 | Sample selection based on a phylogeny of 432 accessions. Phylogenetic tree of 432 accessions, using whole-genome SNPs, based on the Maximum Likelihood method. Wild, *S. candolleanum* (CND) and landrace accessions selected in this study are marked by blue, purple and pink, respectively.

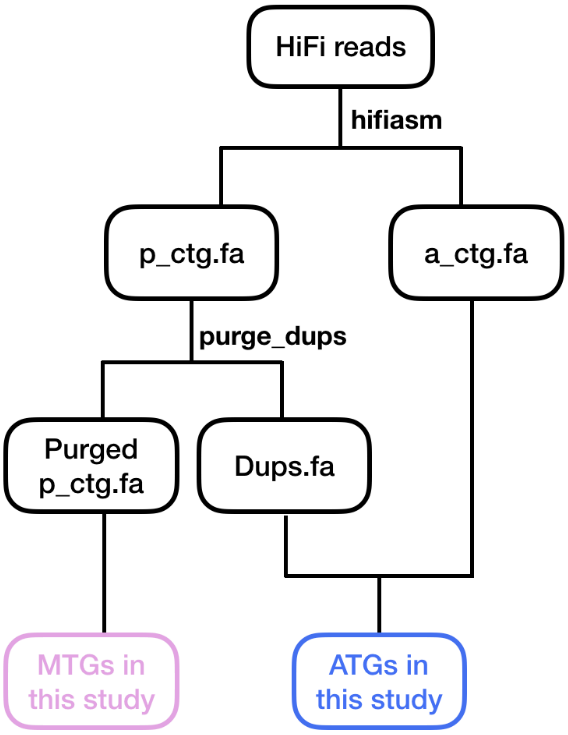

Supplementary Fig. 2 | Overview of the haploidization workflow. PacBio HiFi reads are first assembled by hifiasm, which yields a pair of assemblies: (1) the primary assembly (p_ctg.fa), representing a mosaic haplotype without purging, and (2) the alternate assembly (a_ctg.fa), which represents the alternate haplotype absent from the primary one. Haplotigs are then removed, using the purge_dups software, to generate the monoploid assembled contigs (MTGs), indicative of monoploid genomes. The raw alternate assemblies (a_ctg.fa), in addition to the contigs that have been removed by purge_dups (Dups.fa), were concatenated as the alternate assembled contigs (ATGs) to be the heterozygous genomic segments.

PG1008

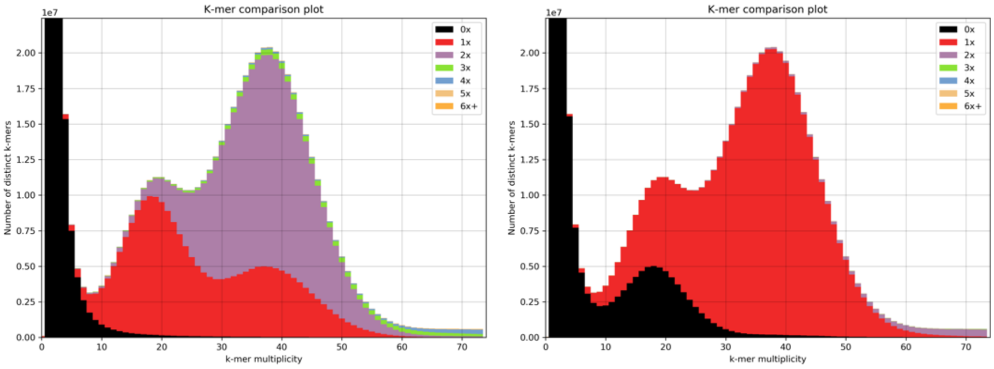

PG1011

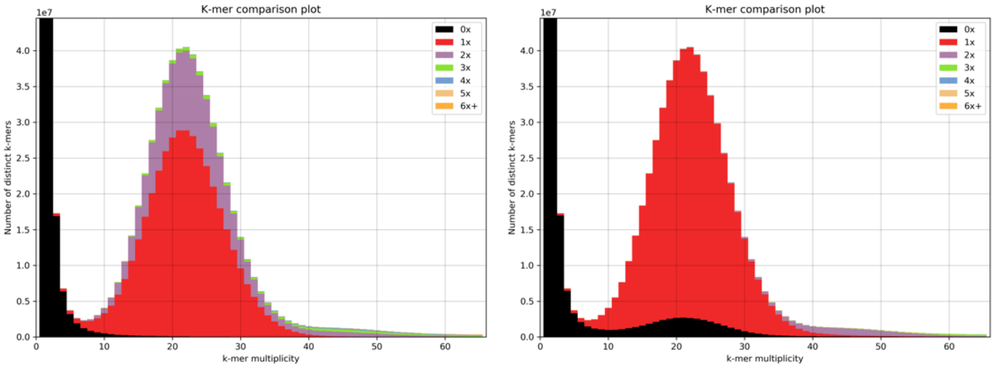

PG1013

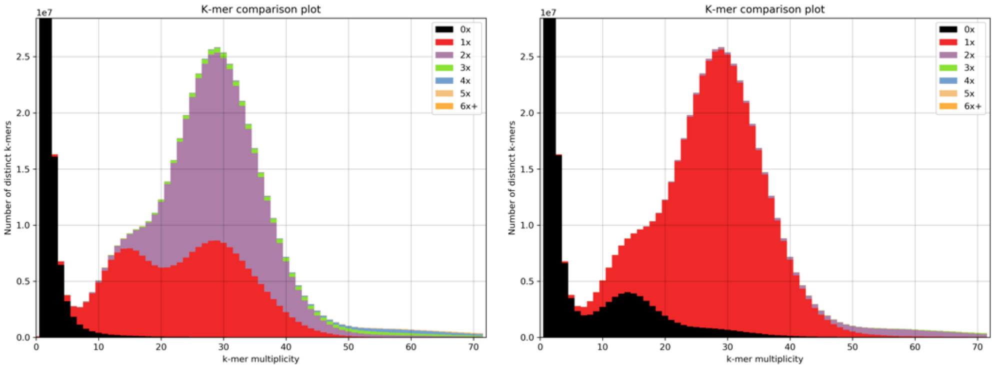

PG3003

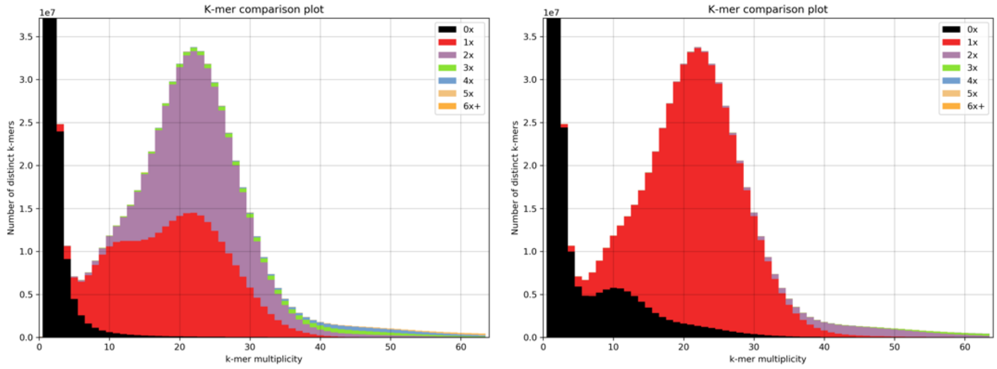

PG3005

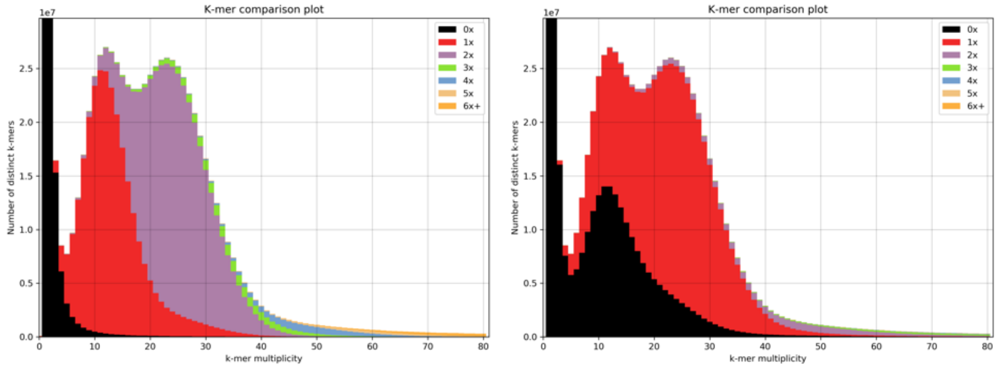

PG3022

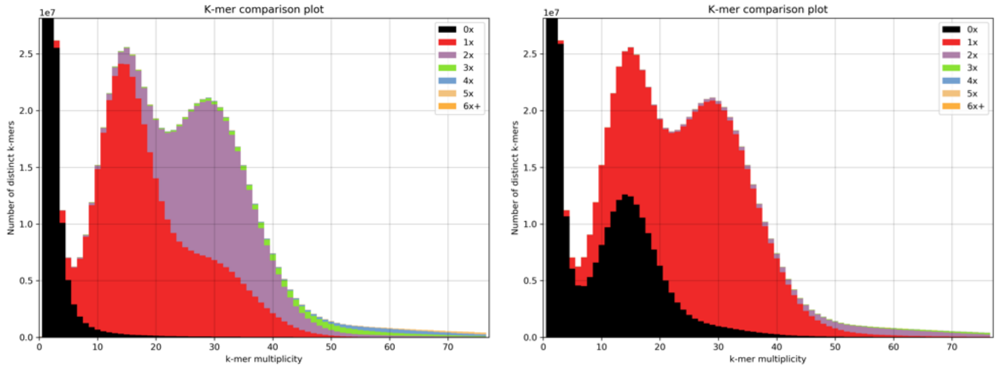

PG3023

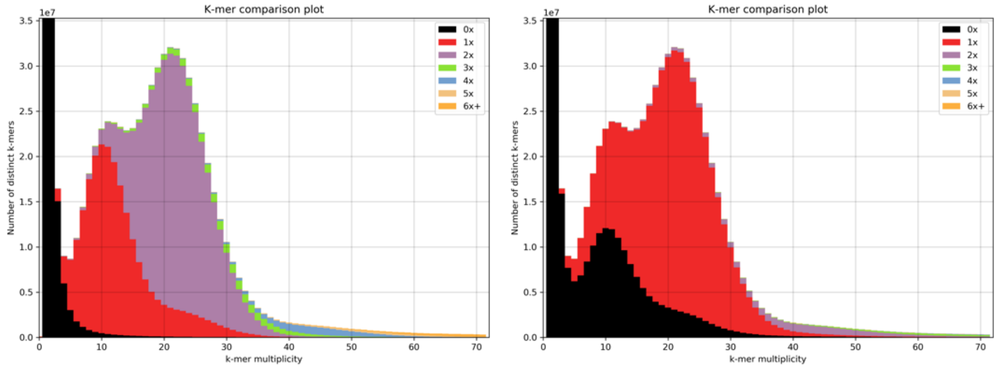

PG4005

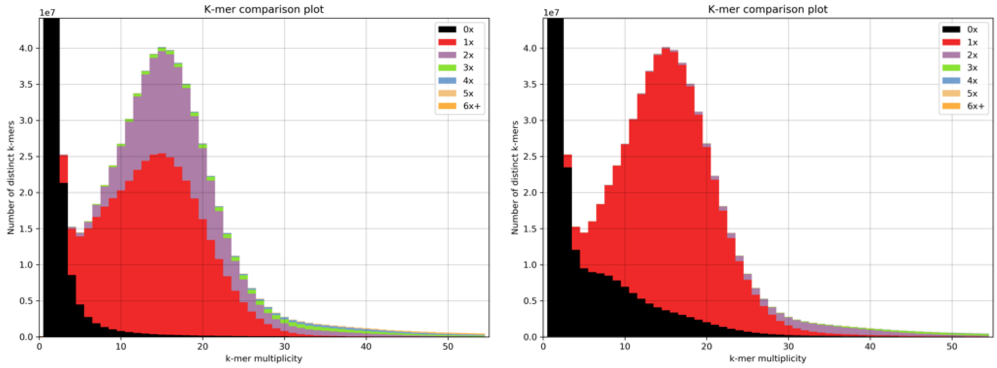

PG4017

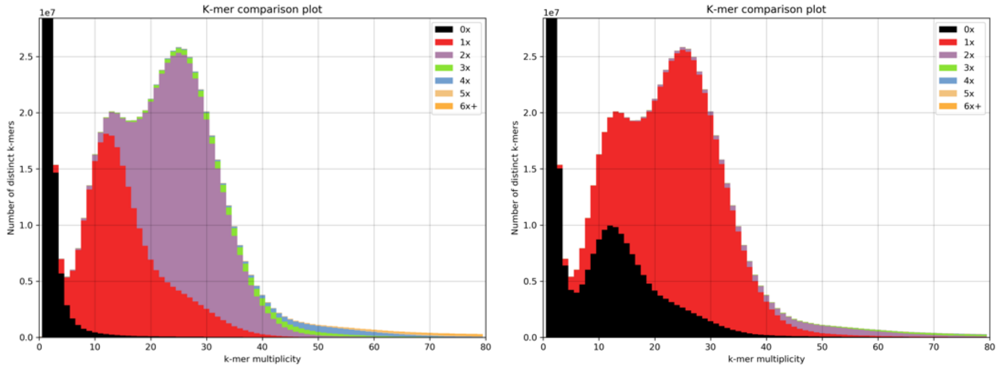

PG4032

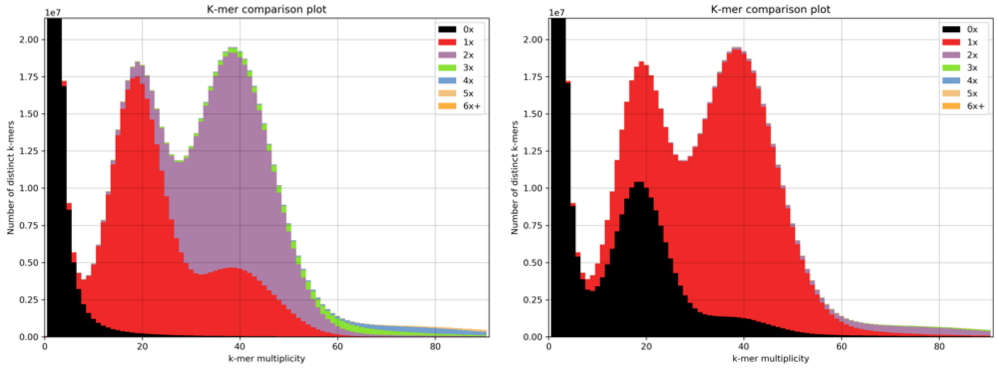

PG4036

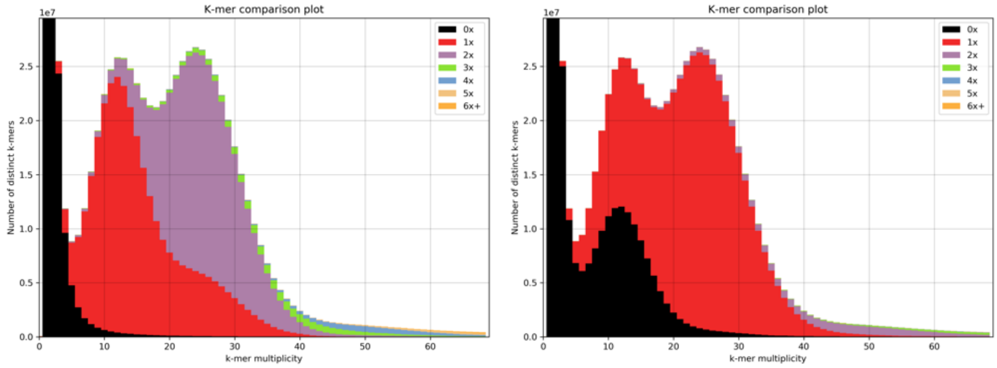

PG4041

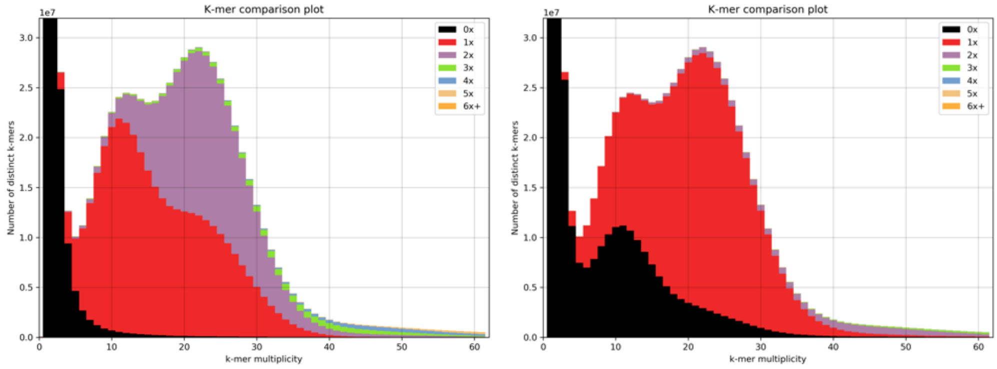

PG4042

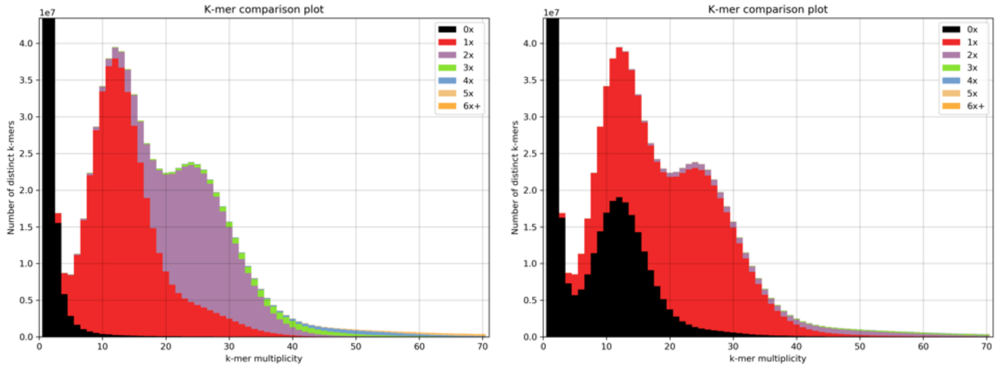

PG4049

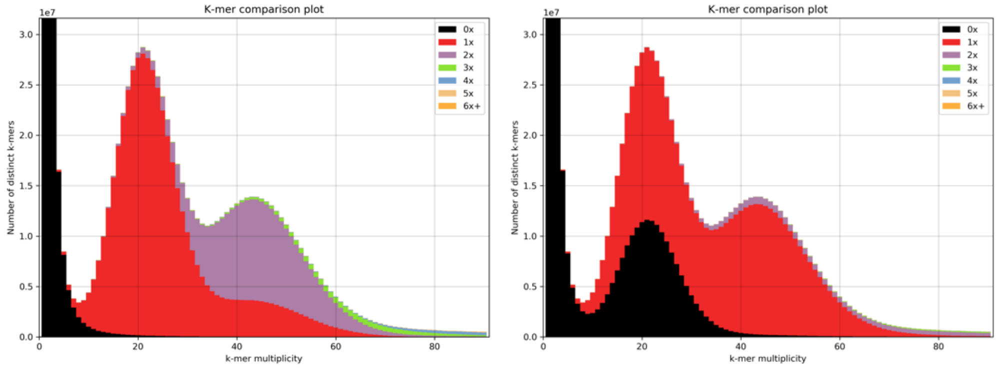

PG4060

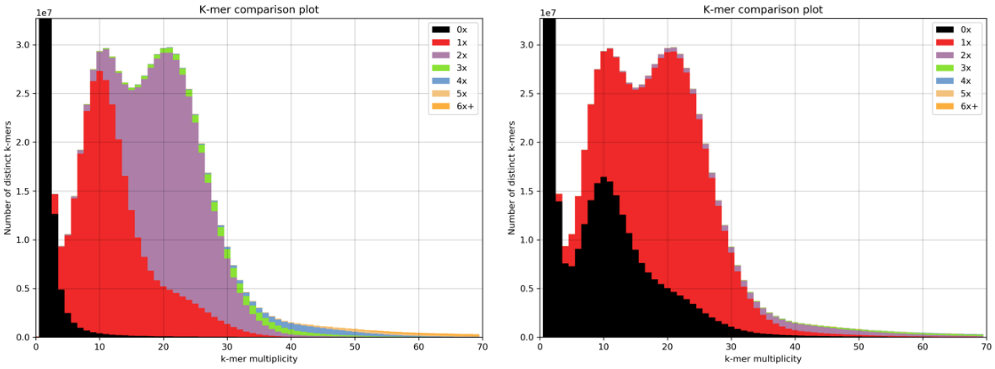

PG5003

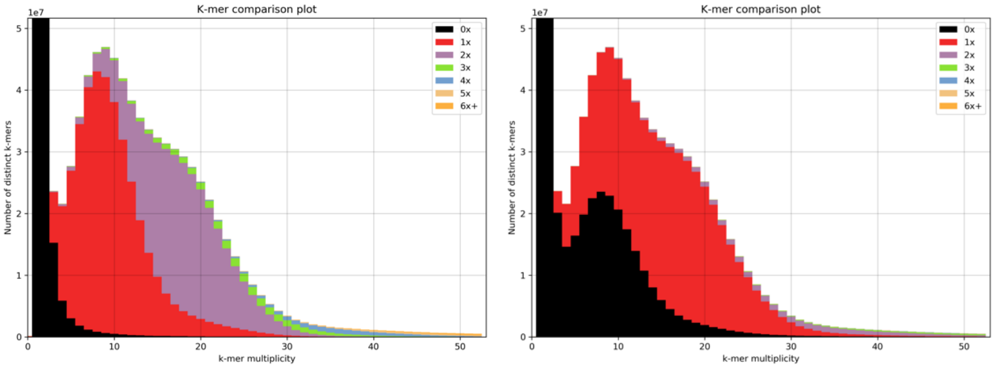

PG5018

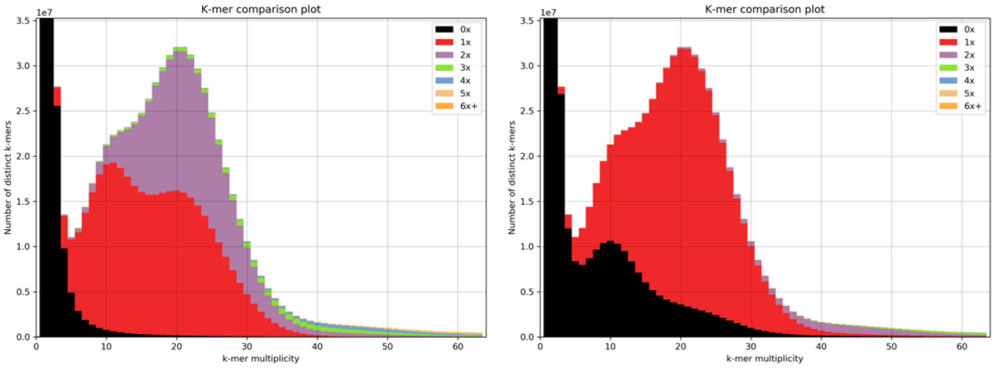

PG5032

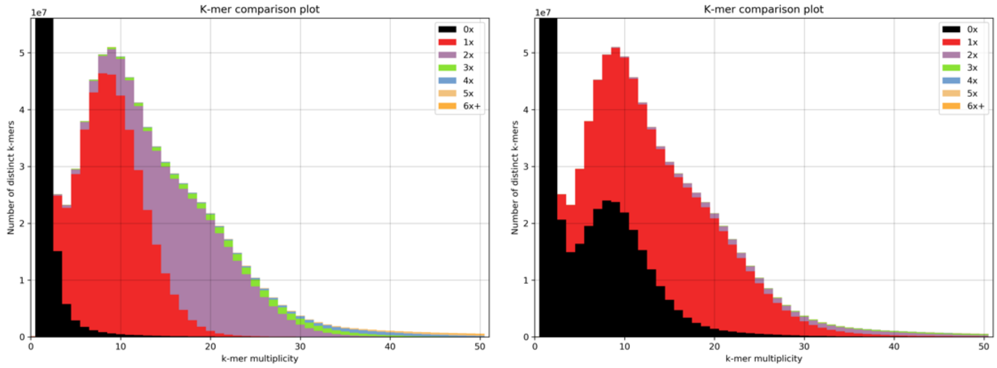

PG5062

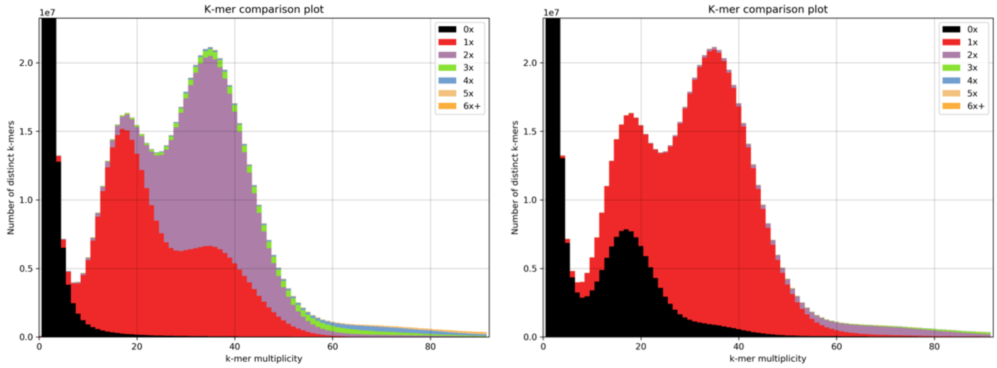

PG5068

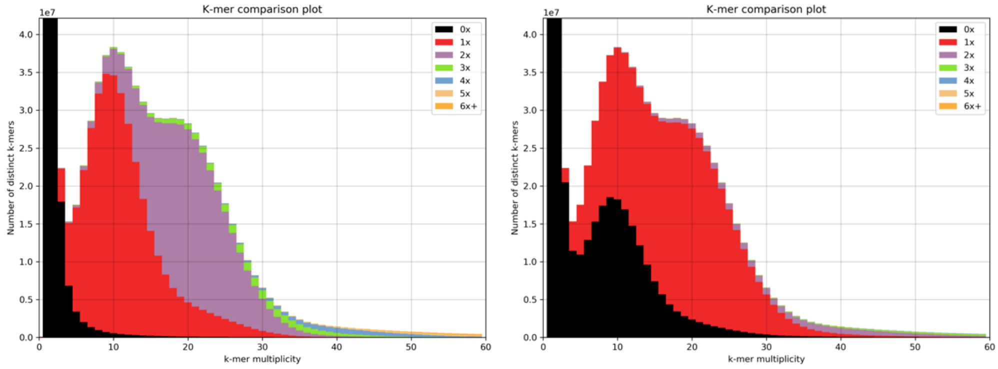

PG5076

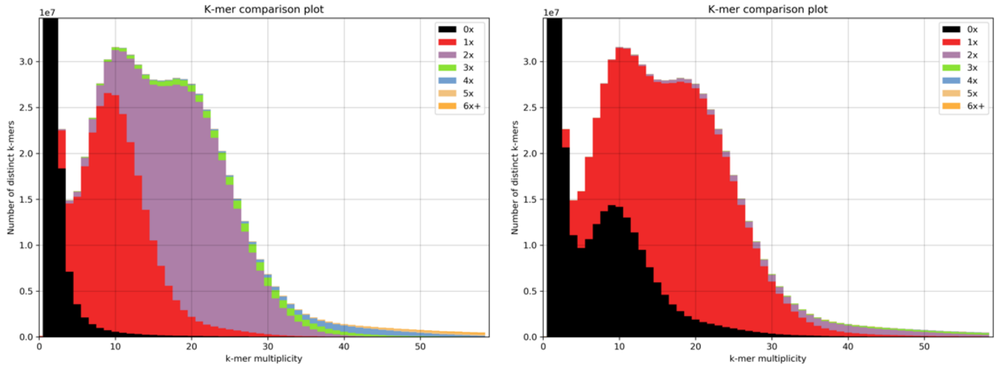

PG6002

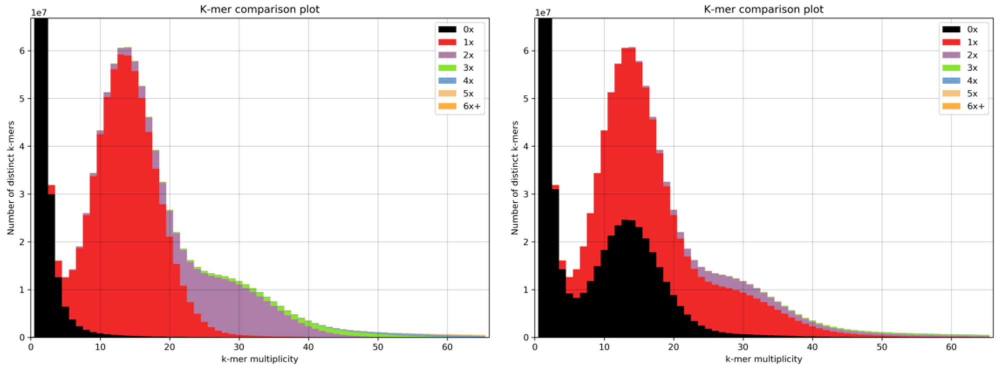

PG6029

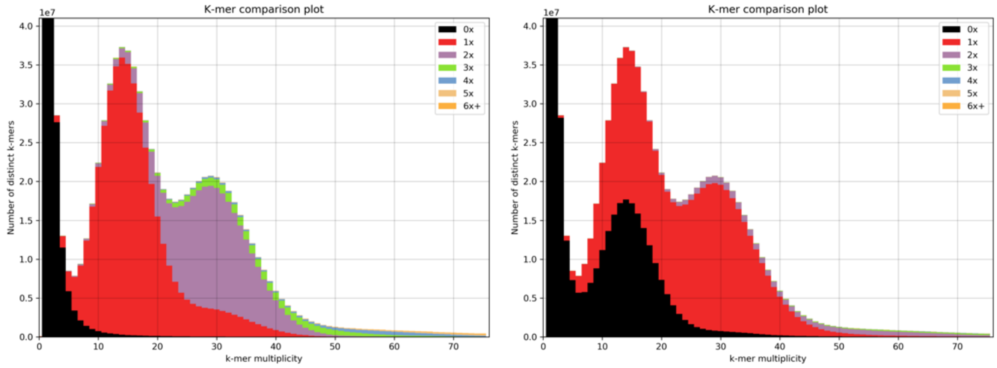

PG6055

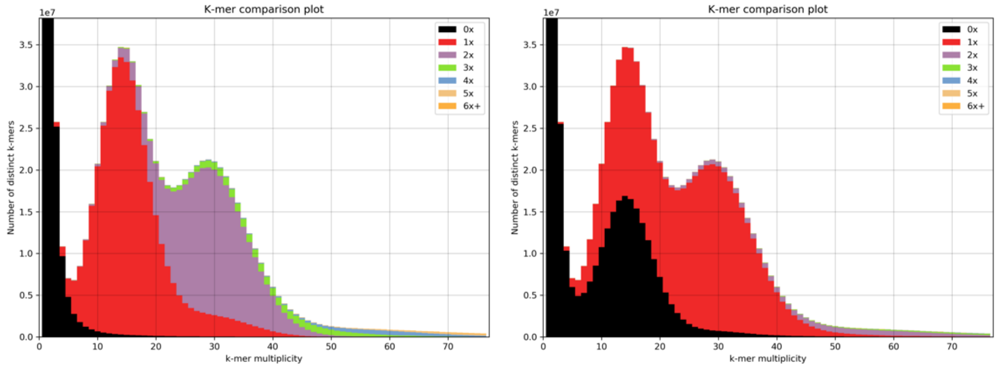

PG6059

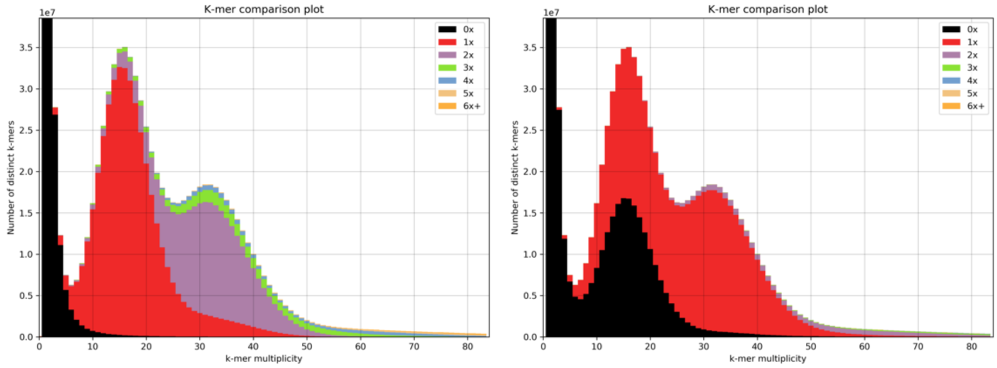

PG6090

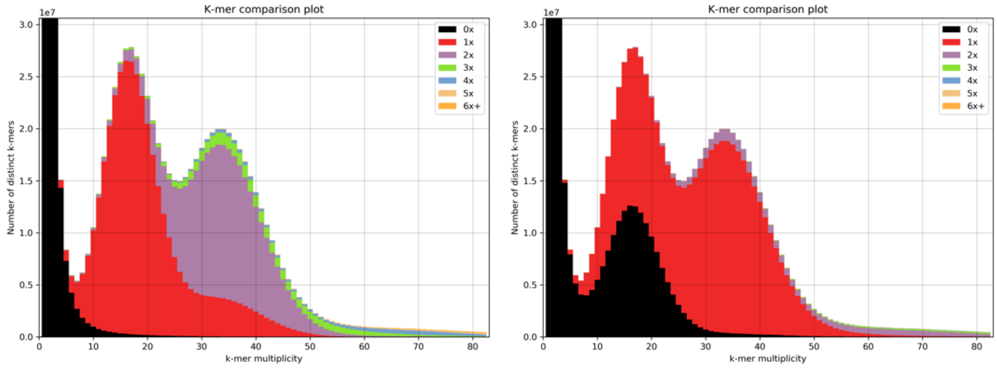

PG6148

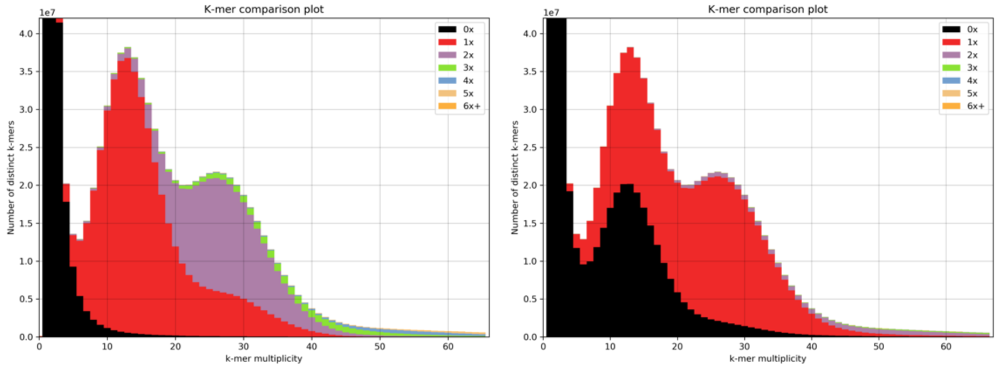

PG6163

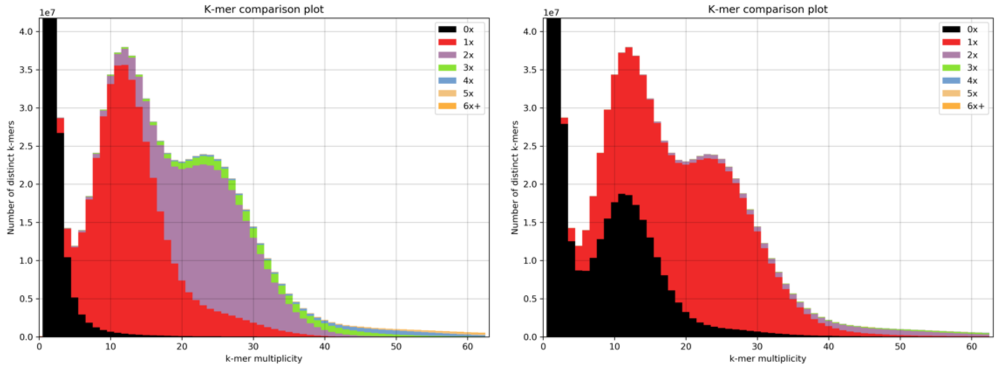

PG6169

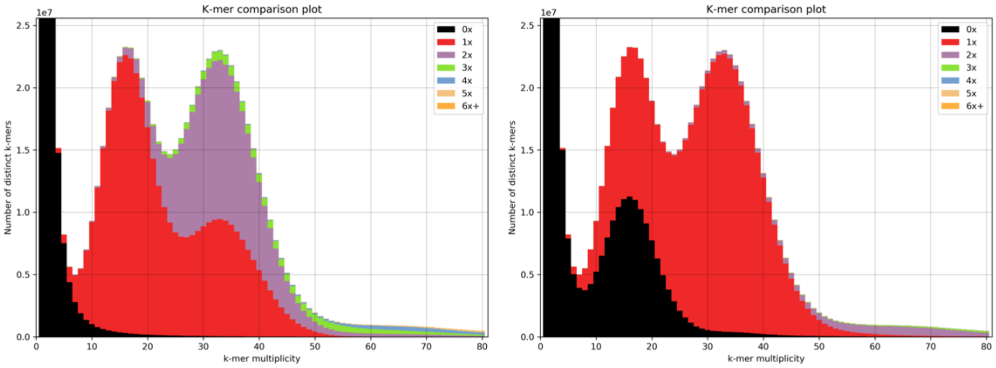

PG6216

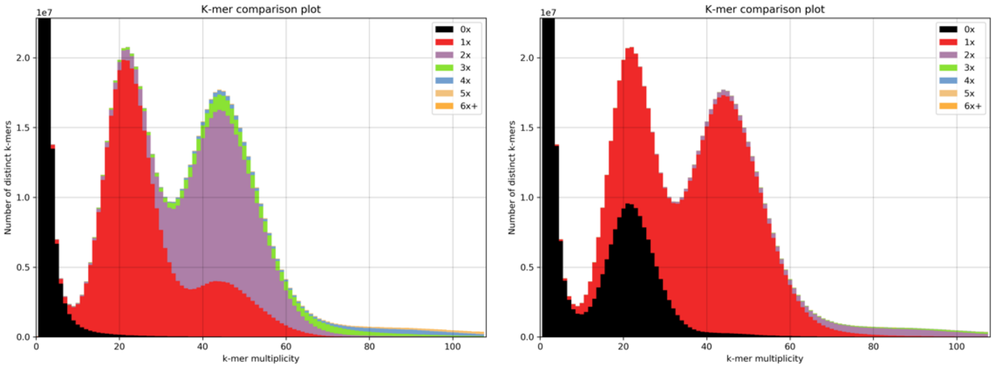

PG6225

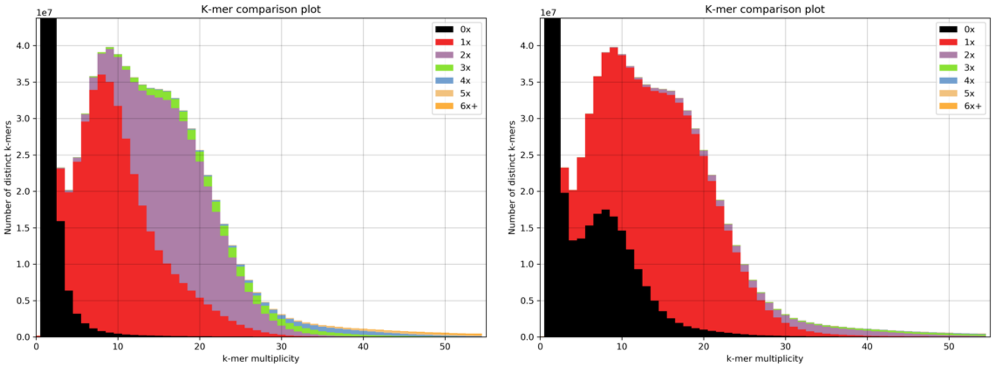

PG6241

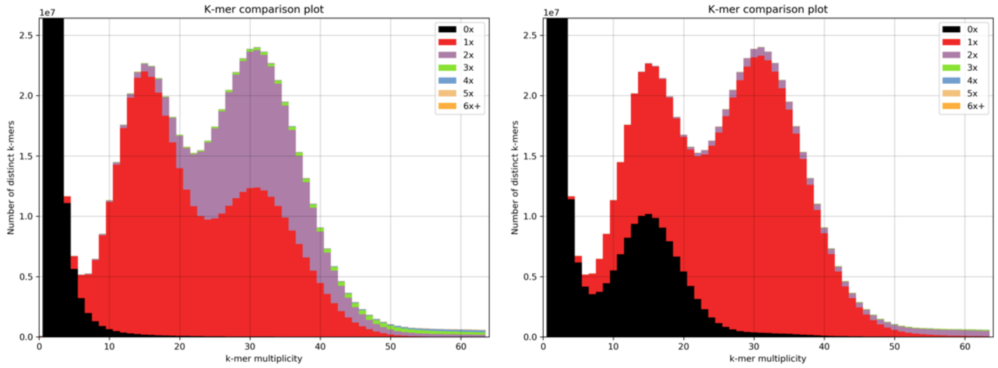

PG6242

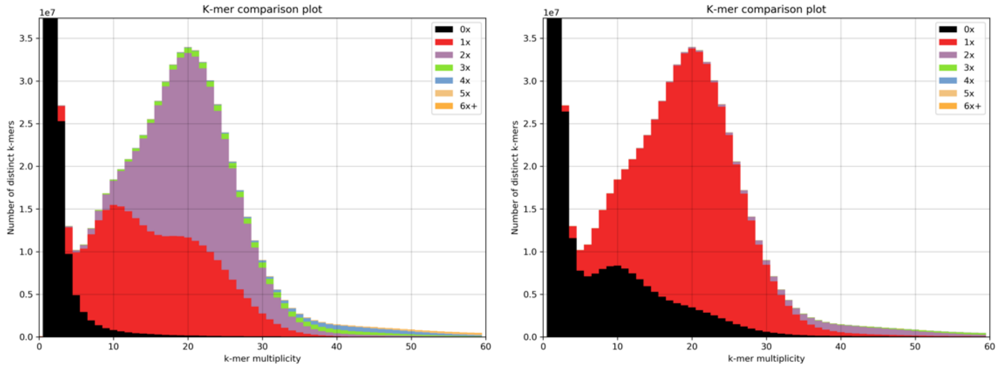

PG6243

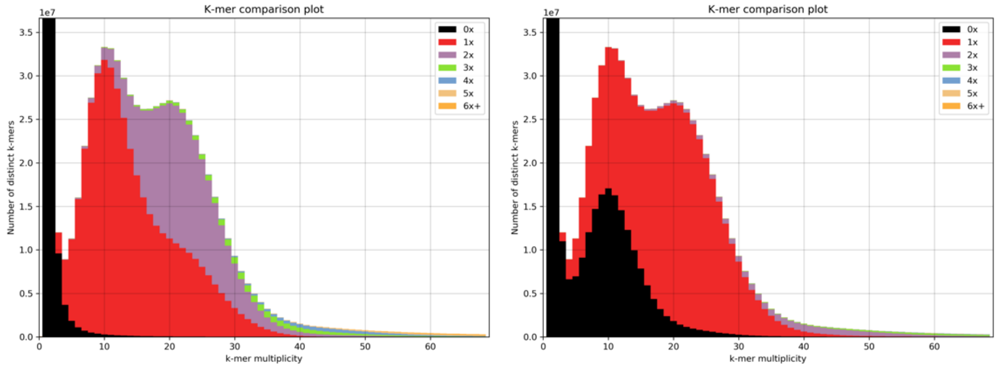

PG6244

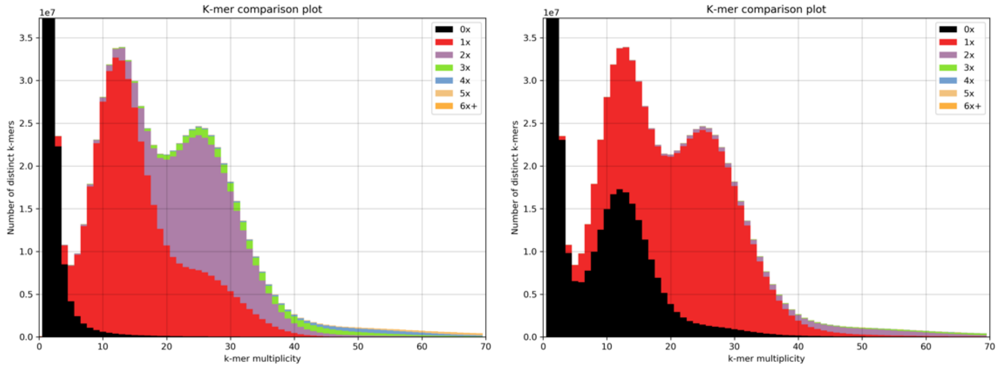

PG6245

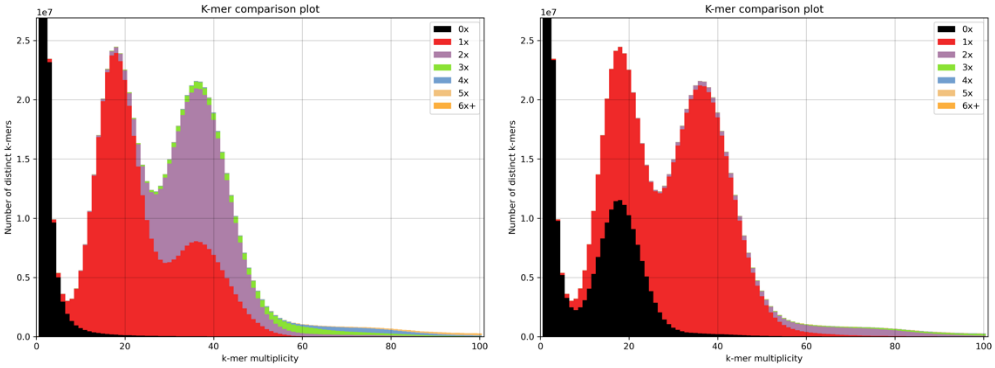

PG6246

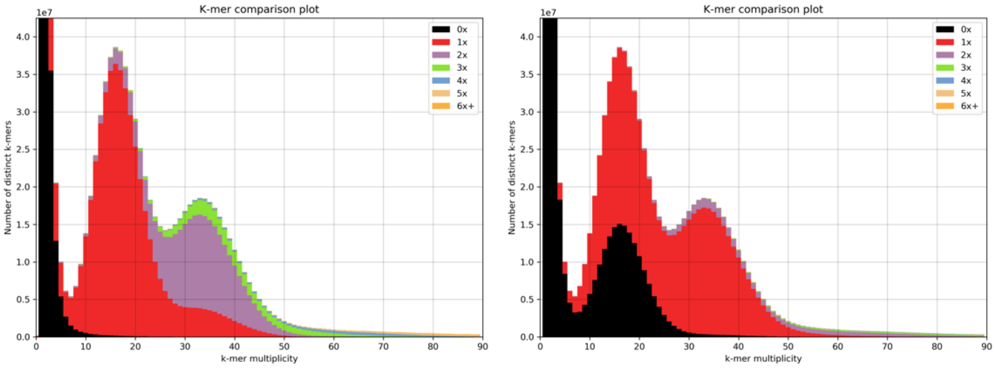

PG6247

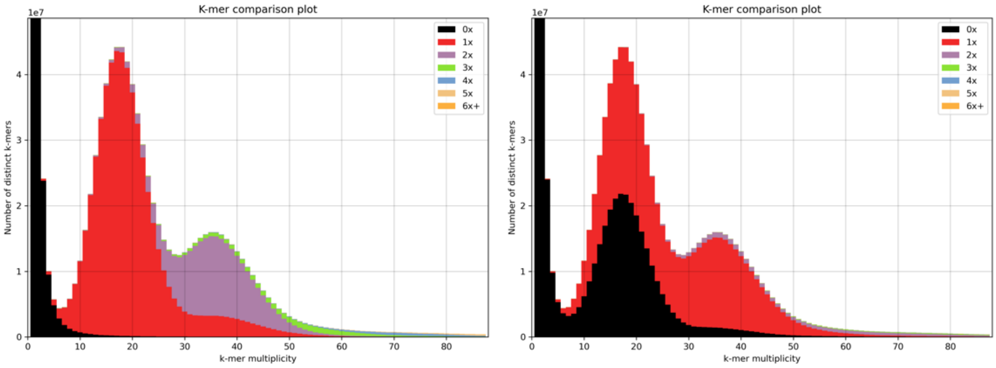

PG6359

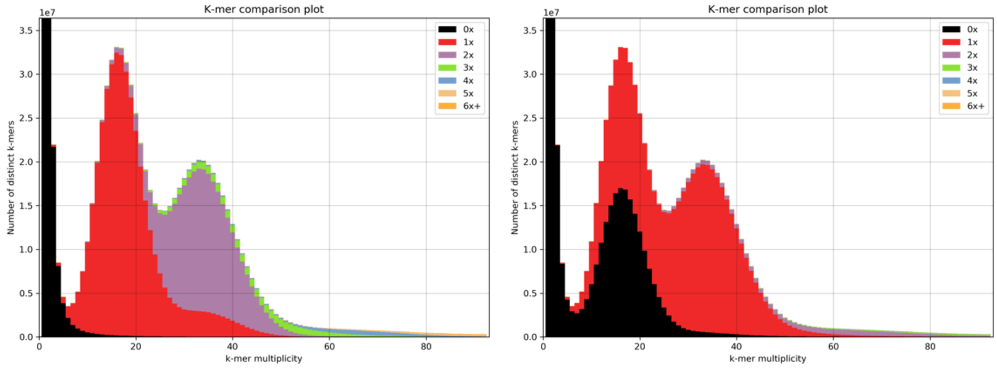

RH10-15

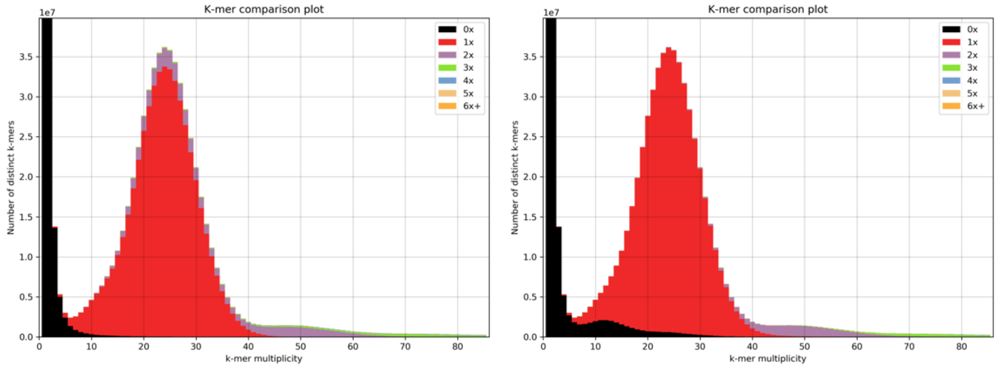

RH

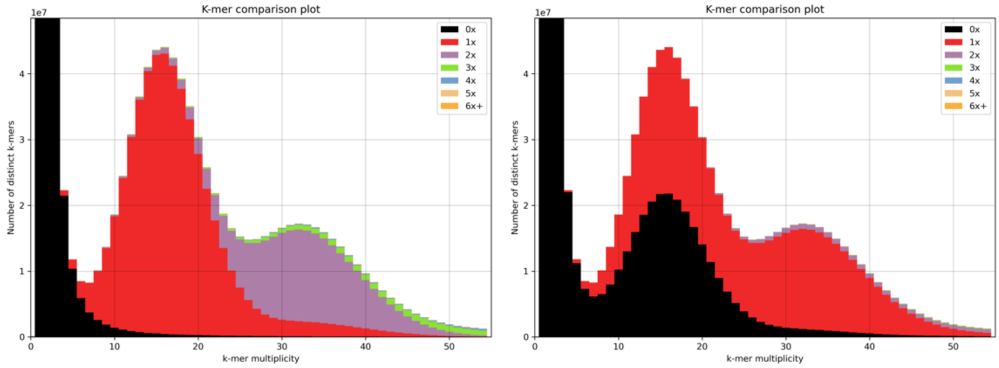

A6-26

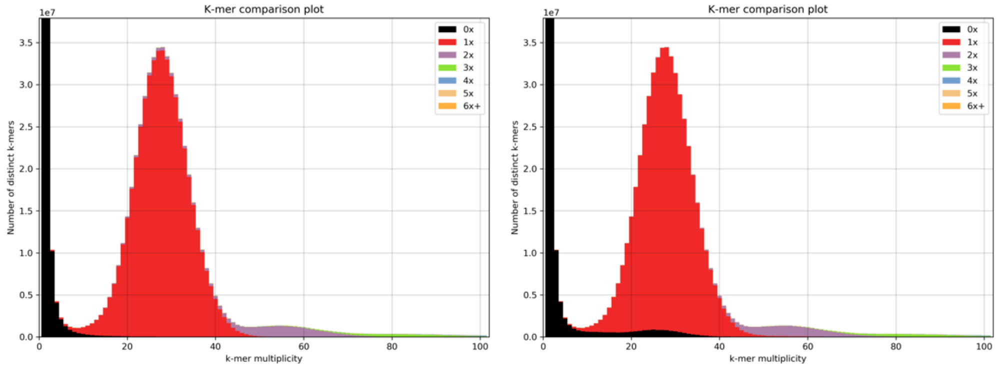

E4-63

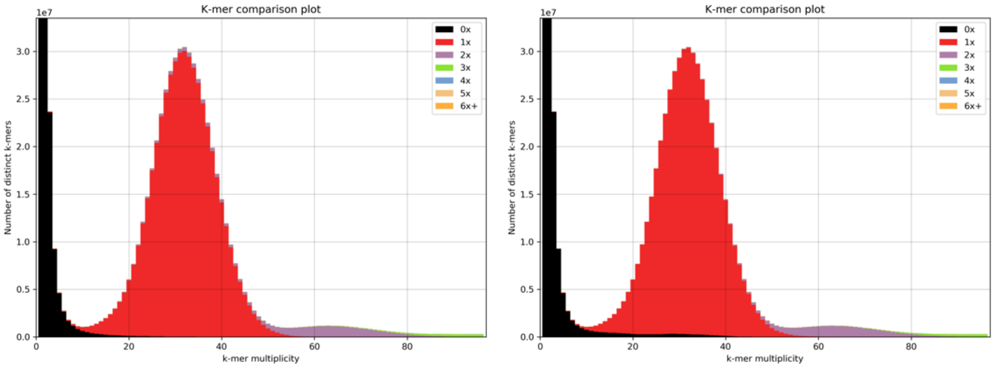

E86-69

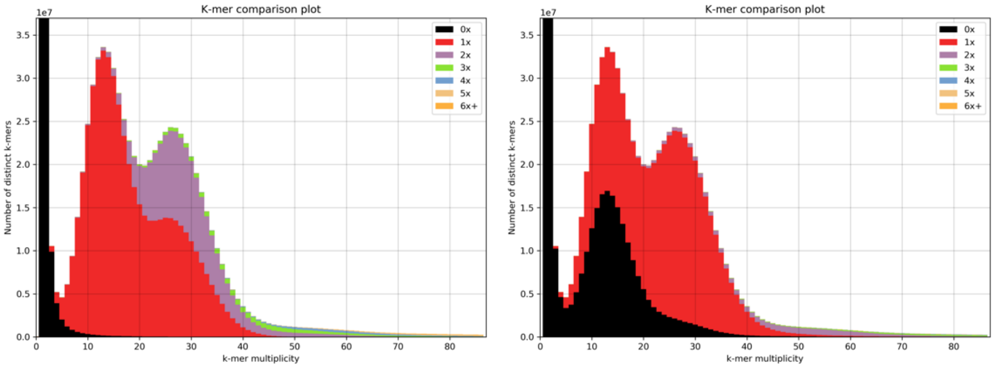

Supplementary Fig. 3 | K-mer spectra assessment for the 44 genome assemblies. For each sample, the left plot shows the 31-mer spectra of the raw assembly, and the right plot depicts the 31-mer spectra of the monoploid assembly. The x-axis indicates the multiplicity of distinct k-mers of raw HiFi reads. The colors represent the times the k-mers were present in the genome assembly. After purging, the heterozygous regions (purple) are mostly collapsed into single-copy homozygous content.

A6-26

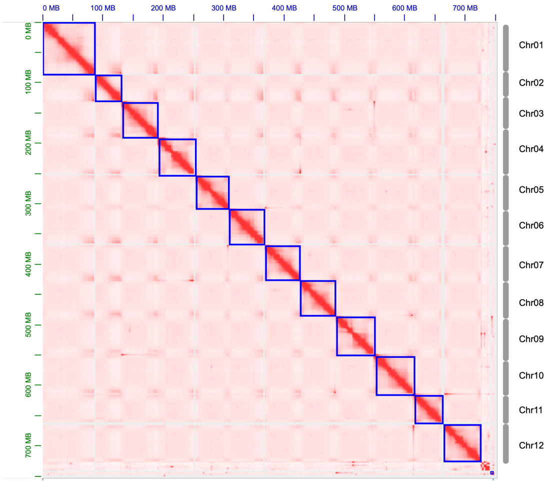

E4-63

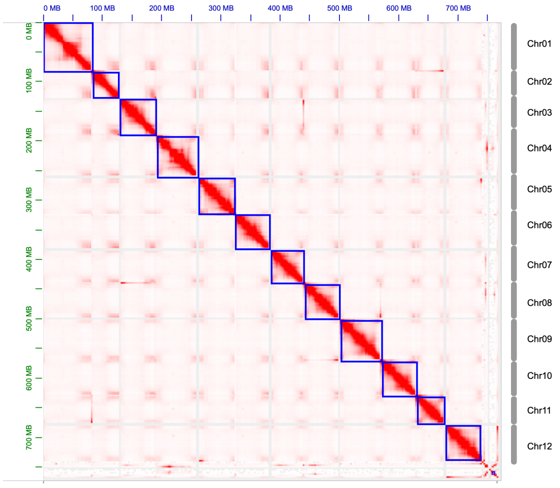

RH10-15

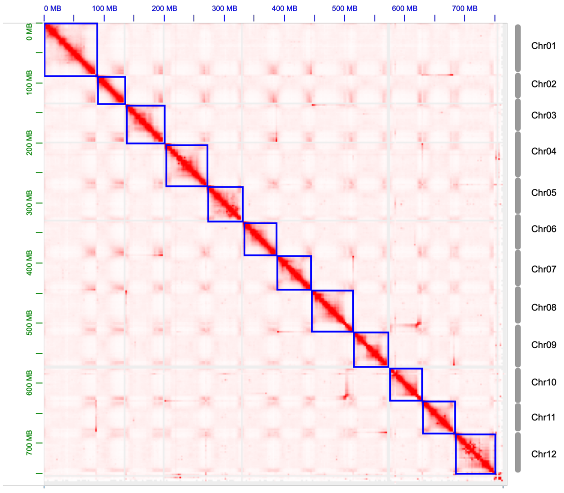

PG6359

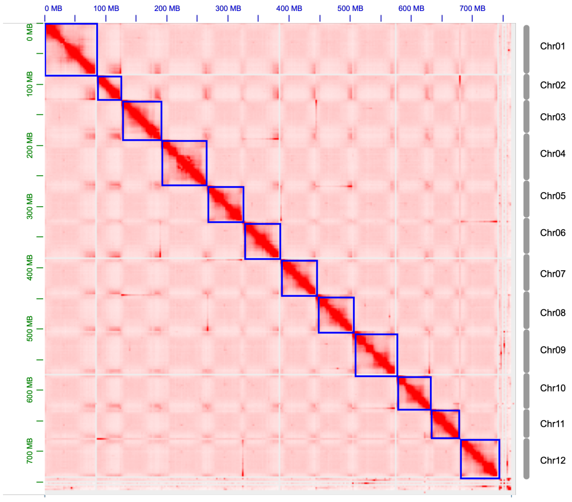

E86-69

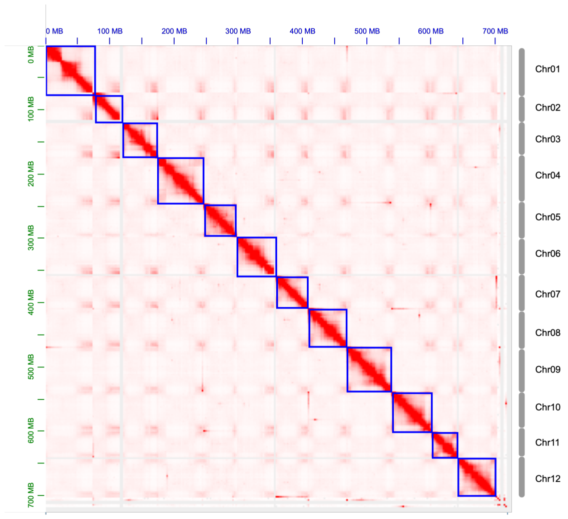

RH

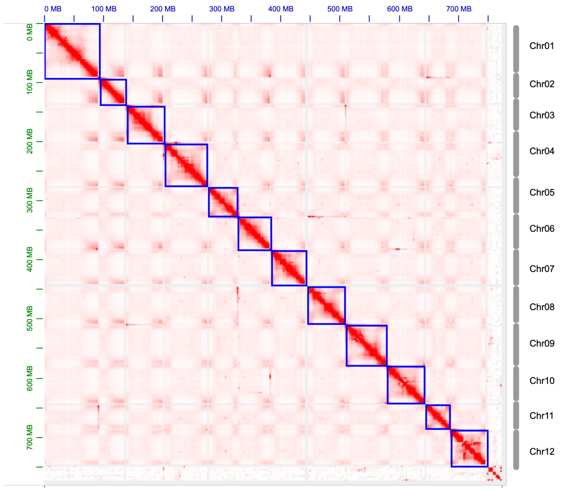

PG5068

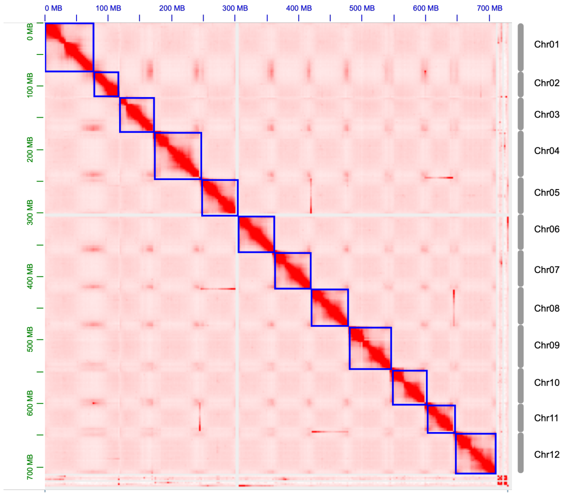

Supplementary Fig. 4 | Hi-C contact matrices of seven pseudochromosome-scale assembled accessions. Heat maps are visualized by Juice box, at 2.5-Mb resolution, to display the whole-genome contact matrices[1]. The order and orientation of the 12 chromosome-scale scaffolds are adjusted using the DM genome as the reference.

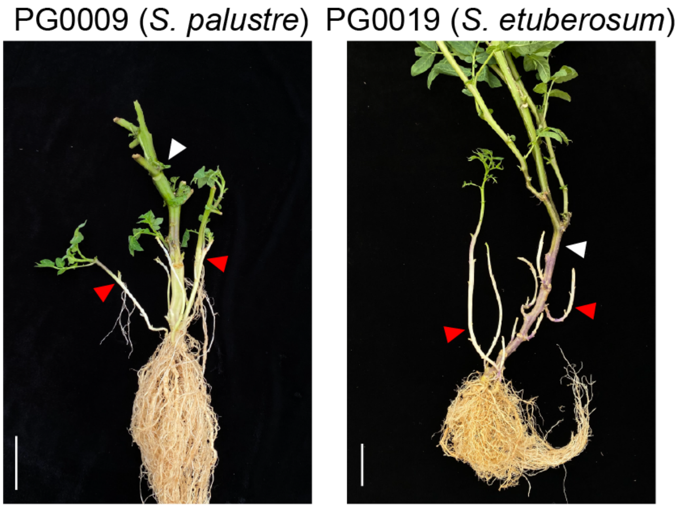

Supplementary Fig. 5 | Phenotypes of rhizomes from *Etuberosum* species. White arrows indicate the plant main stem, and red arrows mark the rhizomes. The plants were grown under long-day conditions for two months. Scale bars: 6 cm.

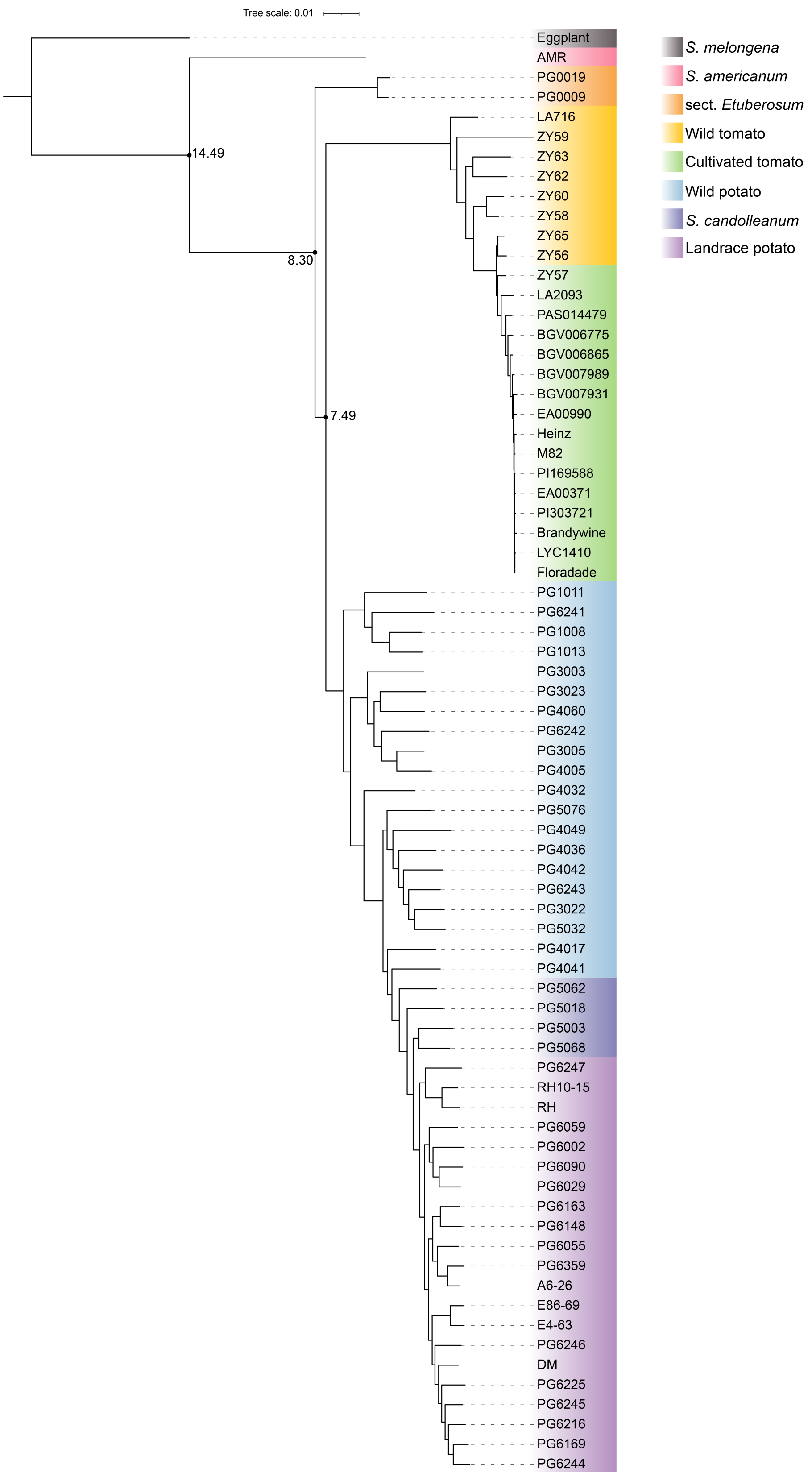

Supplementary Fig. 6 | The estimated divergence time of the 73 accessions used in this study.

Numbers denote the estimated divergence time (million years ago) between key species/clades.

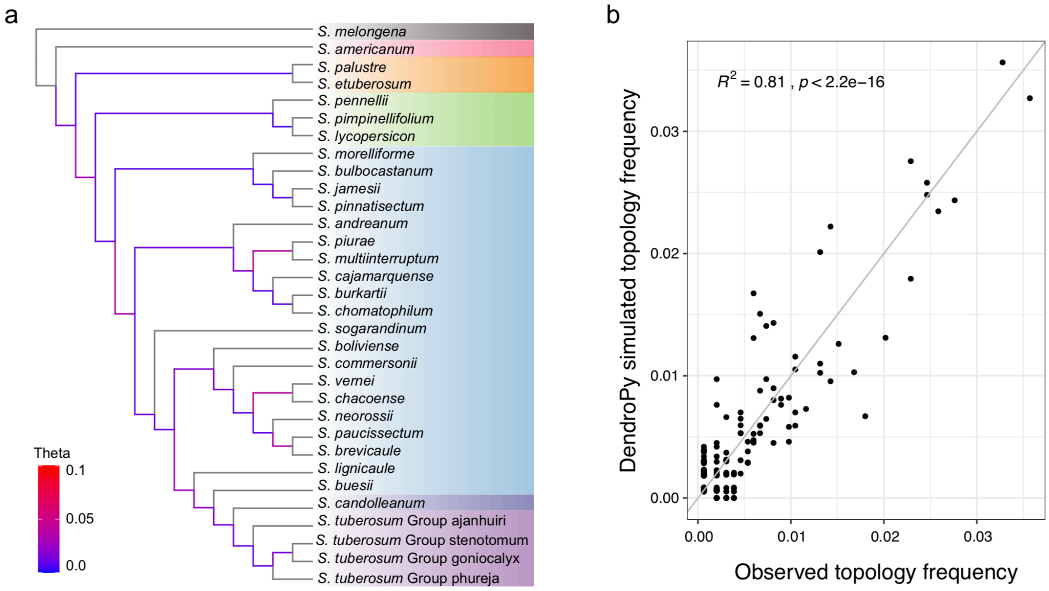

Supplementary Fig. 7 | The impact of ILS on the tree discordance. a, Phylogenetic tree with branches being colored by the inferred population mutation parameter theta, which reflects the population polymorphism, by dividing the mutation units for each internal branch by coalescent units[2]. The grey color denotes branches where theta is unable to be computed due to lack of data. **b**, Simulation of 20,000 gene trees with ILS by DendroPy indicates a positive correlation between the observed and simulated gene-tree topologies from all possible four-species groups among six potato species (*S. tuberosum* Group stenotomum, *S. candolleanum*, *S. lignicaule*, *S. chacoense*, *S. cajamarquense* and *S. bulbocastanum*). The "cor()" function in R was used to perform the correlation analyses, *P*-value < 2.2e-16.

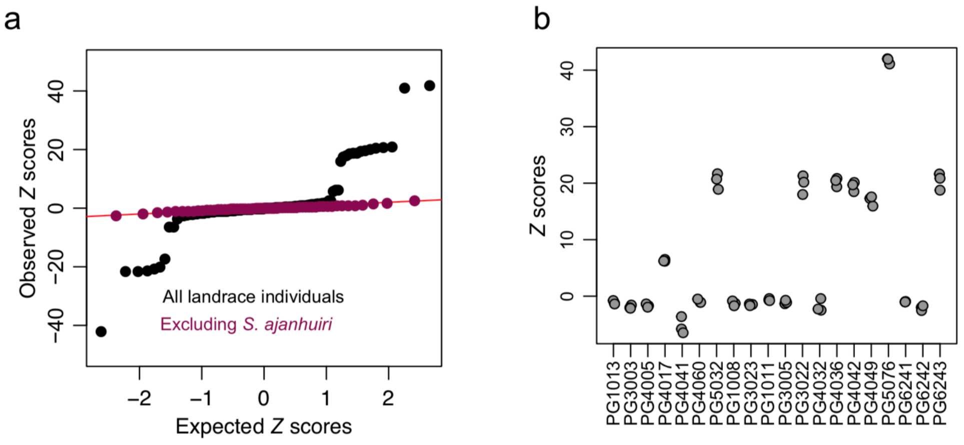

Supplementary Fig. 8 | Gene flow within *Petota*. a, Quantile-quantile plot comparing *Z* scores from all combinations of *D*(landrace, landrace; wild, outgroup) to those expected under a normal distribution. The asymmetric signal is driven by the *S. ajanhuiri* individual (PG6002), which consistently shows closer affinity with wild species than do other landrace accessions. **b,** The *Z* scores for *D*(*S. ajanhuiri*, landrace; wild, outgroup) divided by the wild accessions at the P3 position of the D-statistics along the x-axis. The signal is the strongest when comparing with the *S. boliviense* individual (PG5076) among all tested wild species, suggesting that gene flow has occurred between *S. ajanhuiri* and a sympatric wild species, *S. boliviense*, which is also from Bolivian.

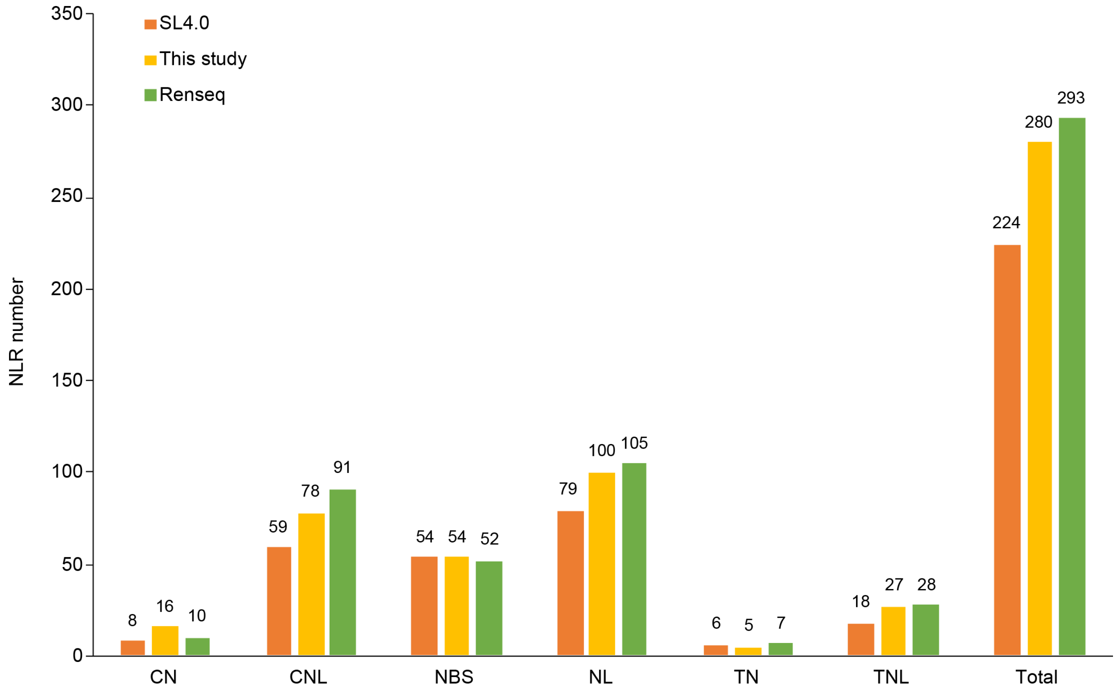

Supplementary Fig. 9 | Comparison of NLR annotation results using the tomato genome (Heinz 1706, SL4.0) as an example. SL4.0: NLRs extracted from its annotation. Renseq: the RenSeq-derived NLR loci reported in Seong *et al.*[3]. CN: CC-NB, CNL: CC-NB-LRR, NBS: NB domain only, NL: NB-LRR, TN: TIR-NB, TNL: TIR-NB-LRR.

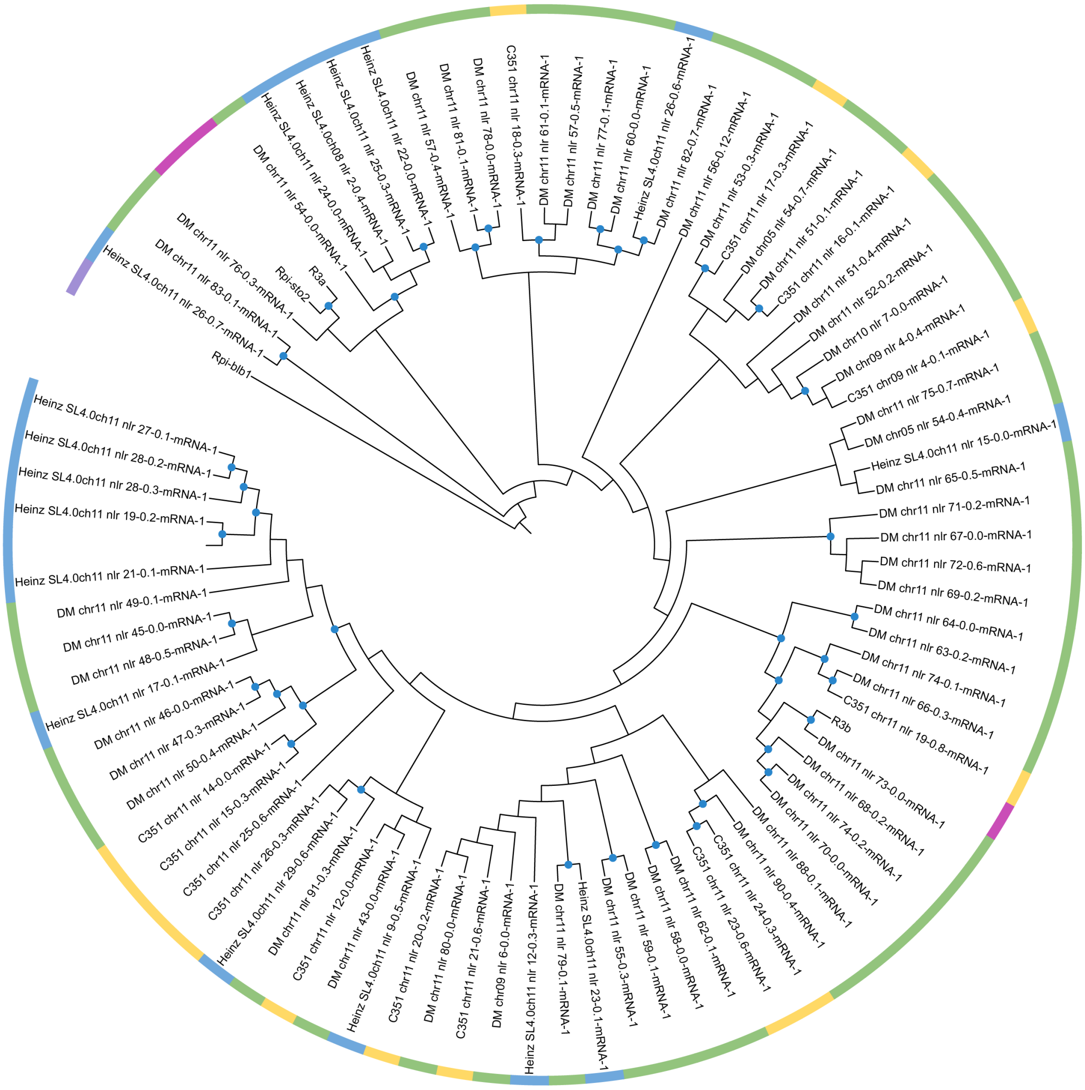

Supplementary Fig. 10 | The phylogeny of the *R3* gene family from DM, Heinz 1706 and PG0019 genomes. The NLRs that classified into *R3* clusters were extracted to infer the phylogeny. Purple: gene serving as outgroup. Blue: NLRs from Heinz 1706 genome. Green: NLRs extracted from DM genome. Yellow: NLRs from *Etuberosum* (PG0019) genome. Wine red: reported functional NLRs. Blue points indicate nodes with bootstrap values ≥ 80.

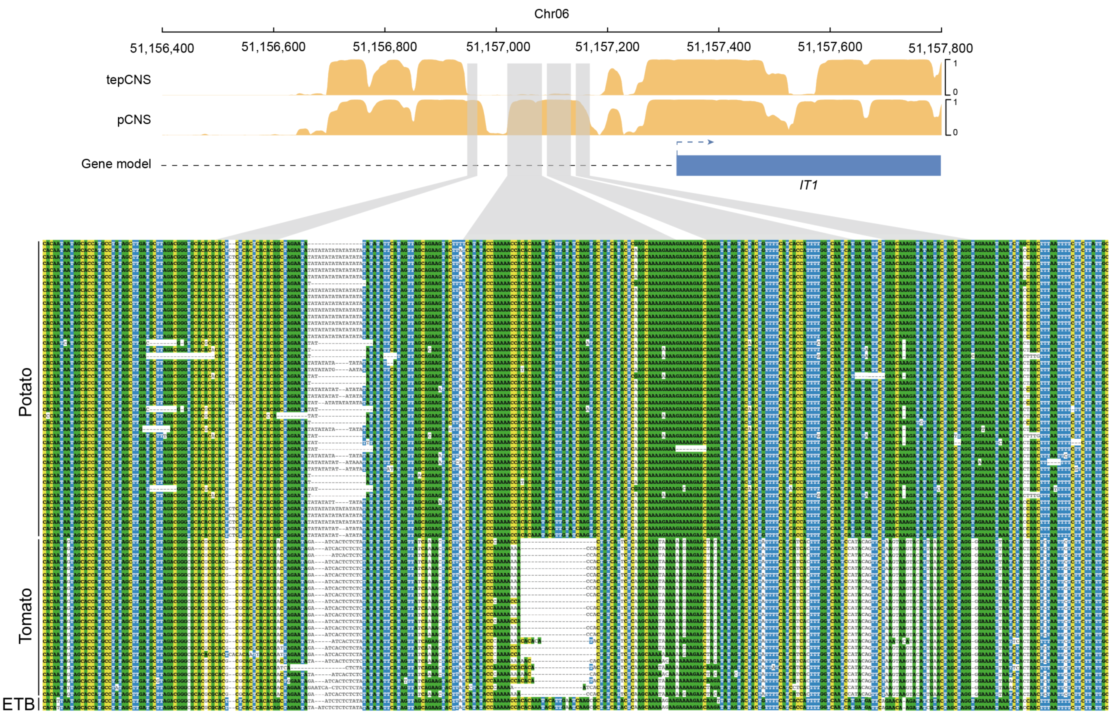

Supplementary Fig. 11 | Alignments of *IT1* associated CNSs. *IT1* associated CNSs alignments among 71 accessions. tepCNS: Conservative scores of each site calculated from tomato, *Etuberosum* and potato genomes; pCNS: Conservative scores of each site calculated from 45 potato genomes; Gray blocks: potato-specific CNSs. ETB: *Etuberosum*.

Supplementary references

1 Durand, N. C. *et al.* Juicer provides a one-click system for analyzing loop-resolution Hi-C experiments. *Cell Syst.* **3**, 95-98 (2016).

2 Cai, L. *et al.* The Perfect Storm: Gene Tree Estimation Error, Incomplete Lineage Sorting, and Ancient Gene Flow Explain the Most Recalcitrant Ancient Angiosperm Clade, Malpighiales. *Syst. Biol.* **70**, 491-507 (2021).

3 Seong, K., Seo, E., Witek, K., Li, M. & Staskawicz, B. Evolution of NLR resistance genes with noncanonical N-terminal domains in wild tomato species. *New Phytol.* **227**, 1530-1543 (2020).
