# Supplementary material for: Genome evolution and diversity of wild and cultivated potatoes
Source: Nature. 2022 Jun 8;606(7914):535–41. doi: 10.1038/s41586-022-04822-x (PMC9200641; doi:10.1038/s41586-022-04822-x)
Supplement: Supplementary file 2 — Reporting Summary [file 41586_2022_4822_MOESM2_ESM.pdf]

nature portfolio

Corresponding author(s): Sanwen Huang

Last updated by author(s): Feb 28, 2022

Reporting Summary

Nature Portfolio wishes to improve the reproducibility of the work that we publish. This form provides structure for consistency and transparency in reporting. For further information on Nature Portfolio policies, see our Editorial Policies and the Editorial Policy Checklist.

Statistics

For all statistical analyses, confirm that the following items are present in the figure legend, table legend, main text, or Methods section.

n/a	Confirmed	
☐	☒	The exact sample size (*n*) for each experimental group/condition, given as a discrete number and unit of measurement
☐	☒	A statement on whether measurements were taken from distinct samples or whether the same sample was measured repeatedly
☐	☒	The statistical test(s) used AND whether they are one- or two-sided<br>*Only common tests should be described solely by name; describe more complex techniques in the Methods section.*
☒	☐	A description of all covariates tested
☒	☐	A description of any assumptions or corrections, such as tests of normality and adjustment for multiple comparisons
☐	☒	A full description of the statistical parameters including central tendency (e.g. means) or other basic estimates (e.g. regression coefficient) AND variation (e.g. standard deviation) or associated estimates of uncertainty (e.g. confidence intervals)
☐	☒	For null hypothesis testing, the test statistic (e.g. *F*, *t*, *r*) with confidence intervals, effect sizes, degrees of freedom and *P* value noted<br>*Give P values as exact values whenever suitable.*
☒	☐	For Bayesian analysis, information on the choice of priors and Markov chain Monte Carlo settings
☒	☐	For hierarchical and complex designs, identification of the appropriate level for tests and full reporting of outcomes
☒	☐	Estimates of effect sizes (e.g. Cohen's *d*, Pearson's *r*), indicating how they were calculated

Our web collection on statistics for biologists contains articles on many of the points above.

Software and code

Policy information about availability of computer code

Data collection

No software was used to collect data. Sequencing platforms used to generate the raw data are listed as followed: PacBio Sequel II, PacBio RS II, Illumina HiSeq X Ten, DNBSEQ-T7.

Data analysis

We used publicly available and appropriately cited software in the Methods. No commercial software and code were used in this study. Software are listed as follows: BWA 0.7.5a-r405, SAMtools v1.9, bcftools v1.9, IQ-TREE v2.0.6, GenomeScope2.0, hifiasm v0.13, purge_dups (v1.01), CANU v1.8, Pilon v1.23, juicer v1.5, 3d-dna v180922, EDTA v1.9.4, RepeatMasker v1.332, HISAT2 (v2.0.1-beta), StringTie (v1.3.3b), BRAKER2 v2.1.6, AUGUSTUS (v3.4.0), GeneMark-ET (v3.67_lic), StringTie (v1.3.3b), MAKER2 (v2.31.11), SNAP (v2013-02-16), InterProScan 5.34-73.0, BUSCO v4.1.4, BLASTP (v2.2.30+), OrthoFinder (v 2.5.2), PanGP (v1.0.1), ParaAT (v2.0), ProgressCactus (v1.2.3), R v4.0.3, PHAST v1.5, DIAMOND (v2.0.6.144), ASTRAL (v5.7.8), MUMmer v4.00rc1, MAFFT (v7.471), BedTools (v2.29.2), PAML v4.9, Dsuite (v0.4 r28), SVIM (v1.4.2), Assemblytics (v1.2.1), SURVIVOR (v 1.0.7), DendroPy (no version), ragtag (v2.1.0), SyRI (v1.4), minimap2 v2.21-r1071, NLR-annotator (v0.7), PRGdb 3.0, RGAugury (v2.2), MView (v1.67), ChIPseeker (v1.24.0) and pyGenomeTracks (v3.6).

For manuscripts utilizing custom algorithms or software that are central to the research but not yet described in published literature, software must be made available to editors and reviewers. We strongly encourage code deposition in a community repository (e.g. GitHub). See the Nature Portfolio guidelines for submitting code & software for further information.

Data

Policy information about availability of data

All manuscripts must include a data availability statement. This statement should provide the following information, where applicable:

- Accession codes, unique identifiers, or web links for publicly available datasets
- A description of any restrictions on data availability
- For clinical datasets or third party data, please ensure that the statement adheres to our policy

All PacBio sequence data, transcriptome data, Hi-C data in this study have been deposited at the National Center for Biotechnology Information (NCBI) Sequence Read Archive (SRA) with BioProject accession number PRJNA754534 and the National Genomics Data Center (NGDC) Genome Sequence Archive (GSA) with BioProject number of PRJCA006011. Genome assemblies, annotation, sequence variation, gene expression for the 46 accessions and the genotype information of 432 lines used for sample selection have been hosted in database the Pan-potato Database (http://solomics.agis.org.cn/potato/, http://218.17.88.60/potato/). Publicly available sequencing data were downloaded from the NCBI with BioProject number of PRJNA641265, PRJNA573826, PRJNA378971, PRJNA394943 and PRJNA766763.

Field-specific reporting

Please select the one below that is the best fit for your research. If you are not sure, read the appropriate sections before making your selection.

☒ Life sciences ☐ Behavioural & social sciences ☐ Ecological, evolutionary & environmental sciences

For a reference copy of the document with all sections, see nature.com/documents/nr-reporting-summary-flat.pdf

Life sciences study design

All studies must disclose on these points even when the disclosure is negative.

Sample size	For pan-genome construction, 44 representative potato accessions were used. These accessions were selected based on their phylogenetic relationship and represented genetic diversity in the potato germplasm.
Data exclusions	No data was excluded.
Replication	Three biological replicates with three technical replicates were used in the qRT-PCR experiment. Three biological replicates were conducted in the yeast-two-hybrid assay. Three independent transgenic knock-out lines were generated for IT1. All replications were successful and were used.
Randomization	Randomization does not directly apply to the genome sequencing and assembly.
Blinding	Blinding does not apply to this study, as the study focuses on comparative genomics and blinding is not necessary.

Reporting for specific materials, systems and methods

We require information from authors about some types of materials, experimental systems and methods used in many studies. Here, indicate whether each material, system or method listed is relevant to your study. If you are not sure if a list item applies to your research, read the appropriate section before selecting a response.

Materials & experimental systems

n/a	Involved in the study
☒	☐ Antibodies
☒	☐ Eukaryotic cell lines
☒	☐ Palaeontology and archaeology
☒	☐ Animals and other organisms
☒	☐ Human research participants
☒	☐ Clinical data
☒	☐ Dual use research of concern

Methods

n/a	Involved in the study
☒	☐ ChIP-seq
☒	☐ Flow cytometry
☒	☐ MRI-based neuroimaging
